# Effects of 28 days of beta-alanine and creatine monohydrate supplementation on muscle carnosine, body composition and exercise performance in recreationally active females

**DOI:** 10.1186/1550-2783-9-S1-P17

**Published:** 2012-11-19

**Authors:** Julie Y Kresta, Jonathan Oliver, Andrew Jagim, Richard Kreider, Jim Fluckey, Steven Reichman, Susanne Talcott

**Affiliations:** 1 Department of Sports Medicine and Nutrition, Human Performance Research Laboratory-Fort Bragg, University of Pittsburgh, Fort Bragg, NC, USA; 2 Department of Health and Kinesiology, Exercise and Sport Nutrition Laboratory, Texas A&M University, College Station, TX, USA

## Background

Early research with beta-alanine (β-ALA) supplementation has shown increases in muscle carnosine as well as improvements in body composition, exercise performance and blood lactate levels. Creatine monohydrate supplementation has been extensively researched for its effects on anaerobic exercise performance. Recently, studies have examined the combined effects β-ALA and creatine supplementation on anaerobic exercise performance and lactate threshold. The purpose of the present study was to examine the acute and chronic effects of β-ALA supplementation with and without creatine monohydrate on body composition, aerobic and anaerobic exercise performance, and muscle carnosine and phosphagen levels in college-aged recreationally active females.

## Methods

Thirty-two females were randomized in a double-blind placebo controlled manner into one of four supplementation groups including β-ALA only (BA), creatine only (CRE), β-ALA and creatine combined (BAC) and placebo (PLA). Participants supplemented for four weeks using an individualized daily dosing strategy that included a loading phase for the creatine for week 1 of 0.3g/kg of body weight and a maintenance phase for weeks 2-4 of 0.1g/kg of body weight, with or without a continuous dose of β-ALA of 0.1g/kg of body weight. They reported for testing at baseline, day 7 and day 28. Testing sessions consisted of a resting muscle biopsy of the vastus lateralis, body composition measurements (DEXA), a graded exercise test on the cycle ergometer for VO_2_max and lactate threshold, and multiple Wingate tests for anaerobic exercise performance.

## Results

Results showed all supplementation strategies increasing muscle carnosine levels over placebo after four weeks, but not between groups. The percent change for each group after four weeks were 35.3±44.8% (p=0.02) for BA, 42.5±99.3% (p=0.01) for BAC, 0.7±27.1% (p=0.04) for CRE versus 13.9±44.0% for PLA. Muscle total creatine showed trends of increasing for all active supplement groups after four weeks, but not between groups. The percent change in muscle creatine after four weeks was 4.6±71.4% for BA, 154.0±375.0% for BAC, 1.7±41.6% for CRE and -4.1±10.9% for PLA (p=0.72). There were improvements for all groups with percent body fat after four weeks (p=0.01), despite the present study not including a specific training protocol. The delta values were -2.3±2.6% BAC, -1.4±4.5% CRE, 0.2±1.8% BA and -1.3±2.2% PLA. There were no group differences observed for VO_2_max (p=0.27), peak lactate (p=0.05) lactate threshold (p=0.67), ventilatory threshold (p=0.35), peak power (p=0.42), mean power (0.28), total work (p=0.28) or rate of fatigue (0.20). There were some trends for anaerobic exercise indicating groups supplementing with creatine may have greater improvements, however, these findings were not statistically significant.

## Conclusions

The present study failed to show any additive effects of β-ALA and creatine supplementation for body composition, aerobic exercise, lactate threshold or anaerobic exercise measures. This could be due to the small sample size resulting in low power and effect sizes. Previous research has demonstrated that four weeks of β-ALA and creatine supplementation was enough time to increase muscle carnosine and phosphagen levels. However, perhaps more time is needed for performance adaptations to occur, especially without the addition of an exercise training component.

